# Performance Evaluation of Commercially Available Masks in Korea for Filtering Airborne Droplets Containing Bacteria

**DOI:** 10.3390/ijerph18157909

**Published:** 2021-07-26

**Authors:** Eun-Hee Lee, Seung-Woo Lee, Seon Young Moon, Jangyup Son

**Affiliations:** 1 Department of Microbiology, Pusan National University, 2 Busandaehak-ro 63 Beon-gil, Geumjeong-gu, Busan 46241, Korea; 2 Department of Fine Chemistry, Seoul National University of Science and Technology, Seoul 01811, Korea; swlee@seoultech.ac.kr; 3 AT-Men Laboratory Co., Ltd., 305 Ho, Jinyang Bldg., 47 Kyonggidae-ro, Seodaemun-gu, Seoul 03752, Korea; symoon8522@gmail.com; 4 Functional Composite Materials Research Center, Korea Institute of Science and Technology (KIST), Wanju-gun, Jeonbuk 55324, Korea; jayson@kist.re.kr; 5 Division of Nano and Information Technology, KIST School University of Science and Technology (UST), Wanju-gun, Jeonbuk 55324, Korea

**Keywords:** woven, fabric, antidroplet, facepiece respirator, Korea filter, bioaerosol

## Abstract

The coronavirus disease (COVID-19) pandemic is a global health threat and has posed a challenge for society and social care services as well as healthcare systems. Due to the risks involved in being exposed to the virus, public health actions such as wearing masks and physical distancing are necessary to reduce its spread. However, using non-validated masks is a serious issue as such masks may provide inadequate protection against airborne bioaerosol transmission, resulting in the spread of the virus. Therefore, it is necessary to evaluate the filtering performances of the masks against bioaerosols as well as particulate matter (PM). Here, we evaluated the filtering performances of sixteen different masks (four brands each of woven, antidroplet, KF80, and KF94 masks) commercially available in Korea with high market shares. As a simulation of being exposed to bioaerosols and to the yellow dust commonly found in Korea, the filtration efficiency levels of the masks were tested against airborne bacteria-containing droplets and against fine dusts of different ranges of particle sizes. Their filtration efficiency levels against the droplets showed strong positive correlations, specifically Pearson correlation coefficient *r* values of 0.917, 0.905, and 0.894, with their efficiency levels against PM1.0, PM2.5, and PM10, respectively. The results of this study should be useful for choosing appropriate masks, including those that meet filtering performance requirements.

## 1. Introduction

Coronavirus disease (COVID-19), caused by severe acute respiratory syndrome coronavirus 2 (SARS-CoV-2), has emerged as a serious global health threat since the first case was identified in Wuhan, China [1,2]. The World Health Organization (WHO) declared COVID-19 a pandemic on 11 March 2020 [3]. COVID-19 has spread globally, and over 2.4 million deaths among 109 million confirmed cases have been reported as of 17 February 2021 [2].

The virus causing COVID-19 spreads easily between people, most commonly through respiratory droplets, small particles, or aerosols [4,5,6]. Thus, airborne transmission of novel coronavirus particles is highly relevant to the spread of the disease. Large droplets (>5 μm in diameter) generally move only a short distance of <1 m; thus, droplet transmission generally occurs in close proximity. Aerosols remain airborne once released in the air and can travel a long distance [1,4]. The WHO has strongly recommended the public wearing of masks, washing hands, and keeping social distance to tackle transmissions of respiratory diseases including COVID-19 [7]. Using personal protective equipment such as a facepiece respirator (FPR) is also of great importance for providing protection from infectious diseases.

Even prior to COVID-19, Koreans frequently experienced yellow dust and generally used masks to protect their respiratory organs from the inhalation of fine dusts in polluted air. Particulate matters (PMs) have been classified as PM1.0 (<1 μm), PM2.5 (<2.5 μm), and PM10 (<10 μm) based on their particle sizes [8]. PMs can, when inhaled, cause serious health problems, such as nonfatal heart attacks and bronchial asthma. The International Agency for Research on Cancer (IARC) of the WHO has classified PMs as a Group I carcinogen. In this regard, the Korean government has established standards for testing filtration efficiencies or penetration levels of masks against PMs. The Korean government also encourages the use of certified Korea filter (KF) masks for preventing the transmission of respiratory diseases.

Many studies have reported the filtration performances of facepiece respirators against PMs or bioaerosols based on national or international standards, and they were mostly performed prior to the COVID-19 situation [9,10,11,12]. Guidelines for masks in Korea are currently based on tests of their filtration capabilities using sodium chloride (NaCl) and paraffin oil and carried out for a period of 30 s [13]. Brochot et al. evaluated filtration efficiency of filtering facepiece respirators (FFRs) against NaCl particles ranging from 20 to 600 nm [12]. However, these guidelines do not necessarily match the way masks are used in real-life situations, in large part since they are actually worn for much longer than 30 s.

Choosing an appropriate mask is very important for protecting the user of the mask—and others—from the transmission of infectious diseases. KF (Korea filter) masks (KF80, KF94, and KF99), approved by the Korea Ministry of Food and Drug Safety, have been widely used to filter bioaerosols as well as PMs in Korea. Antidroplet and fabric masks have also been commonly used for preventing airborne transmission of bioaerosols despite a lack of any guarantee of their filtration performances. Therefore, it is necessary to evaluate the filtration capabilities of the masks against airborne bioaerosols as well as against fine dust for a prolonged period of time.

In this study, we evaluated the filtration performances of sixteen different masks (four brands each of woven, antidroplet, KF80, and KF94 masks) commercially available in Korea with high market shares. To simulate real-life exposure to yellow dust and airborne bioaerosols, the filtration capabilities of the masks were tested using fine dust (PM1.0, PM2.5, and PM10) and using airborne droplets containing bacteria. The relationships between the filtration efficiencies of the masks against the PMs and their filtration efficiencies against airborne bacteria-containing droplets were also evaluated. The surface of each tested mask was examined using scanning electron microscopy (SEM) and energy-dispersive X-ray spectroscopy (EDS) analysis.

## 2. Materials and Methods

### 2.1. Filtration System Used for the Performance Evaluation

**Figure 1:**
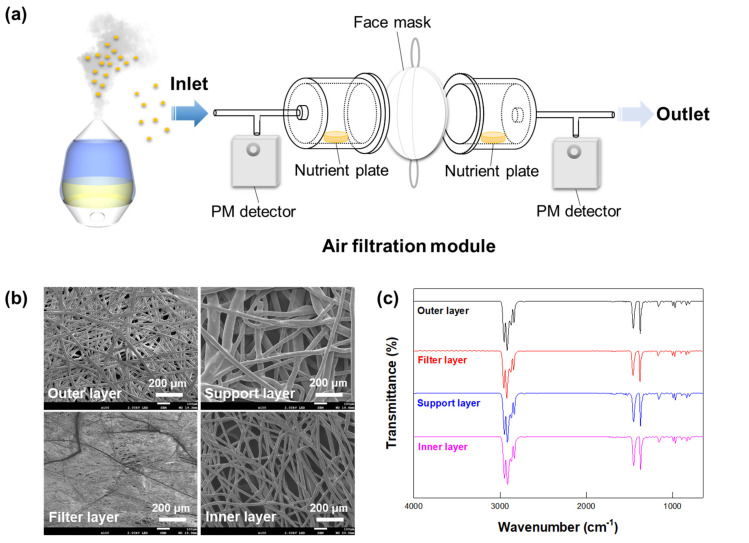
(**a**) Schematic illustration of the filtration system used to evaluate the effectiveness levels of masks against PMs and airborne bacteria-containing droplets. (**b**) Scanning electron microscopy (SEM) images and (**c**) Fourier-transform infrared spectroscopy (FT-IR) spectra of the various layers of a KF80 mask (category A).

The filtration system used to evaluate the performances of the masks mainly consisted of an inlet, filtration module, and outlet (Figure 1a). The inlet port was connected to a chamber with a dimensions of 100 cm × 60 cm × 120 cm (width × length × height), and generated contaminated air was made to flow through the inlet from the chamber. The filtration module was composed of two custom-made acrylic cylinders (7.5 cm × 10 cm, diameter × length), and two O-rings were inserted between them. Each facet of the cylinder had a 1/4″-diameter hole and silicone tubing was connected to each hole. A face mask was placed between the cylinders of the filtration module, and the cylinders were tightly closed using an aluminum flange clamp. Thus, no leakage occurred during the operation. A vacuum pump was connected with silicone tubing to the outlet port. PM detectors (PM2008M, Cubic Sensor and Instrument Co., Ltd., Wuhan, China) were placed in the inlet and outlet ports, respectively, and were linked to a computer that automatically recorded concentrations of PM1.0, PM2.5, and PM10.

### 2.2. Selection of Masks Commercially Available in Korea

Sixteen different face masks commercially available in Korea—four each of woven, antidroplet, KF80, and KF94 masks—were selected for the performance evaluation (Table S1). Woven masks, having diverse designs, are more flexible and air permeable than the other masks, and there are no specific market requirements to sell woven masks. Antidroplet masks are generally light, air permeable, and effective in blocking the transmission of droplets. The KF masks classified as health masks are intended for preventing the passage of particulate matters (PMs) such as yellow dust and are certified by the Korea Ministry of Food and Drug Safety. The number next to the KF mark indicates the percentage of particles that the mask can prevent from passing through. For example, KF80 means a greater than 80% filtration capability against fine dust when it tested using particles with dimensions of 0.6 μm, and KF94 shows higher than 94% of filtration against particles with dimensions of 0.4 and 0.6 μm. Antidroplet masks have a flat shape, while others show a cup shape (Table S1). The selected non-woven masks were label certified. All of the masks used in the current study were purchased, and done so easily, either online or at physical sites in Korea.

### 2.3. Filtration Capability of the Masks against Particulate Matters (PMs)

As a simulation of yellow dust or fine particles, PMs were produced by fuming incense (Perak, Malaysia) into the chamber. Air containing PMs was introduced into the filtration module using a vacuum pump at a flow rate of 30 L/min according to the KMFDS testing protocol [13]. The filtration system was continuously operated for 30 min. The concentrations of PM1.0, PM2.5, and PM10 particles were measured every second using PM detectors at the inlet and outlet ports. The filtration efficiency of each mask against PM was calculated using the equation

$$\mathrm{Filtration}\;\mathrm{efficiency}\;\mathrm{against}\;\mathrm{particulate}\;\mathrm{matter}\;(\%)=(1-\frac{C_{\mathrm{outlet}}}{C_{\mathrm{inlet}}})\times 100$$

where *C_inlet_* and *C_outlet_* represent PM concentrations (μg/m_air_^3^) at the inlet and outlet, respectively. Each of the experiments was performed at least in triplicate unless otherwise indicated.

### 2.4. Filtration Performances of Masks against Airborne Bacteria-Containing Droplets

The Gram-positive bacterium *Bacillus subtilis* (FBCC-B1550, Nakdonggang National Institute of Biological Resources, Gyeongsangbuk-do, Korea) was used in this study. *Bacillus subtilis* is a spore-forming organism that can exist and survive in the form of bacterial cells and spores in the air [14,15]. The bacterium *Bacillus subtilis* was cultured in nutrient broth (MBcell, KisanBio Co., Seoul, Korea) at 30 °C with shaking of 180 rpm for 16 h. The culture broth was centrifuged to collect the cells at 5000 rpm for 10 min, and washed with phosphate-buffered saline (PBS, pH 7.0, Sigma-Aldrich, St. Louis, MO, USA) solution twice. A bacterial suspension at a concentration of 5.0 ± 1.4 × 10^7^ colony forming units (CFU)/mL was loaded into an atomizer (DP3355-UH, Yangil Co., Gyeonggi, Korea). The airborne *Bacillus subtilis*-containing droplets were generated by atomizing a washed culture broth of 10 mL. Nutrient agar plates (Duchefa Biochemie, Haarlem, The Netherlands) were placed inside the filtration unit near the inlet and outlet, and were used to collect airborne bacteria-containing droplets at these locations (Figure 1a). After this collection, the plates were incubated at 30 °C for 16 h, and then photographed. Colonies grown on the plates were counted, and the efficiency of each mask at filtering airborne droplets containing bacteria was measured by comparing CFUs of the plates at the inlet and outlet locations.

$$\mathrm{Filtration}\;\mathrm{efficiency}\;\mathrm{against}\;\mathrm{airborne}\;\mathrm{bacteria}-\mathrm{containing}\;\mathrm{droplet}\;(\%)\;=(1-\frac{{\mathrm{CFU}}_{\mathrm{outlet}}}{{\mathrm{CFU}}_{\mathrm{inlet}}})\times 100$$

### 2.5. Scanning Electron Microscopy (SEM) and Energy-Dispersive X-ray Spectroscopy (EDS) Analysis

Each mask was cut into 1 cm × 1 cm pieces. Such a piece of the mask was coated with a thin layer (~100 nm) of platinum and then subjected to surface analysis. The specimen to be analyzed was viewed employing a field-emission scanning electron microscope (FE-SEM, JSM-7900F, JEOL Ltd., Tokyo, Japan), with an accelerating voltage of 2 kV. The elemental mapping of the surface of the mask was performed by carrying out EDS analysis using an AZtec Xmax-170 apparatus (Oxford Instruments, Abingdon, UK).

### 2.6. Fourier-Transform Infrared Spectroscopy (FT-IR) Analysis

An FT-IR spectrum of each layer of each analyzed mask was collected using a Vertex 80v (Bruker, Ettlingen, Germany) FT-IR spectrometer equipped with an IR scope (Hyperion, Bruker) from 4000 to 650 cm^−1^ with a resolution of 4 cm^−1^. Each spectrum represented an average of 512 scans.

## 3. Results and Discussion

### 3.1. Properties of the Commercially Available Face Masks

The filtration capabilities of a total of 16 brands of masks were evaluated against PMs and airborne bacteria-containing droplets (Table S1). The woven masks only included one layer of fabric with a thickness of 1.0 to 1.5 mm. These fabrics, made of polyester, polyurethane, and spandex, showed skeins of thread each with a thickness of about 12 μm (Figure S1).

The non-woven masks were each composed of three to four layers, namely outer, filter, and inner layers with or without a support layer (Table S1). As shown in Figure 1b, each layer of the non-woven KF80 mask consisted of entangled fibers of various thicknesses. The outer layer was formed from fibers with diameters of 15–25 μm, while the support layer was made of fibers with diameters of 13–27 μm. The filter layer was formed from thinner fibers, with diameters of only 2.0–4.5 μm, while the inner layer was composed of relatively thick fibers with diameters of 20–36 μm. As a result, the filter layer was a fine-meshed net, while the support layer was a wide mesh. Despite their different mesh forms, all of the mask layers showed identical FT-IR spectra, each consisting of ten distinct IR absorption bands at 2952, 2917, 2838, 1455, 1376, 1166, 997, 973, 840, and 808 cm^−1^ (Figure 1c), which were assigned to polypropylene [16]. These spectroscopy results indicated that all of the layers of the non-woven masks were made of only polypropylene.

### 3.2. Efficiencies of the Masks at Filtering Air Particulate Matters (PMs)

**Figure 2:**
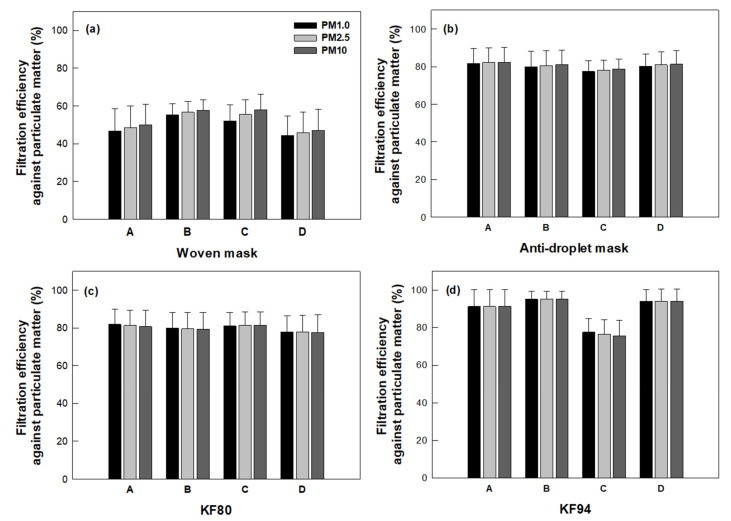
Efficiency levels of commercially available face masks at filtering air particulate matters (PMs) (*n* = 3). (**a**) Woven, (**b**) antidroplet, (**c**) KF80, and (**d**) KF94 masks. A, B, C, and D indicate categories of the masks described in Table S1.

Different face masks showed different filtration efficiencies against PMs (Figure 2). The woven masks showed the lowest average filtration efficiencies, specifically from 44.3 ± 10.4 to 55.4 ± 5.8% against PM1.0, 45.8 ± 10.8 to 56.7 ± 5.7% against PM2.5, and 47.0 ± 11.3 to 57.9 ± 8.2% against PM10 (Figure 2a). The antidroplet masks recorded average filtration efficiencies of 77.5 ± 5.7 to 81.7 ± 8.1% against PM1.0, 78.1 ± 5.4 to 82.1 ± 7.9% against PM2.5, and 78.7 ± 5.2 to 82.3 ± 7.9% against PM10 (Figure 2b). The KF80 masks achieved similar filtration efficiencies as did the antidroplet masks, specifically 78.0 ± 8.5 to 81.9 ± 7.9% against PM1.0, 77.9 ± 8.8 to 81.4 ± 8.2% against PM2.5, and 77.7 ± 9.4 to 81.6 ± 7.0% against PM10 (Figure 2c).

The KF94 masks, belonging to the categories A, B, and D, showed the highest average filtration efficiencies of all the masks tested, with values of 91.2 ± 9.0 to 95.1 ± 4.1% against PM1.0, 91.3 ± 8.9 to 95.1 ± 4.0% against PM2.5, and 91.4 ± 9.0 to 95.2 ± 4.1% against PM10 (Figure 2d). Unexpectedly, compared to these masks, the other tested category of KF94 masks (category C) showed considerably lower average filtration performances, specifically a filtration efficiency of 77.6 ± 7.2% against PM1.0, 76.5 ± 7.7% against PM2.5, and 75.6 ± 8.3% against PM10 (Figure 2d).

SEM images showed that PMs were filtered by all the mask layers (Figure S2). Irrespective of the mesh size of the mask layer, diverse sizes of PMs, specifically with dimensions of 0.1–10 μm, were observed on the surface of each layer. Moreover, the KF80 mask without a support layer (i.e., category C) showed a filtration efficiency insignificantly different from those of the other KF80 masks that did each contain a support layer (i.e., categories A, B, and D).

Other studies have also reported the poor performances of cloth masks [17,18,19,20,21]. Rengasamy et al. reported a much higher penetration of aerosols through cloth masks than through N95 respiratory filter masks [17]. Jung et al. reported that handkerchiefs appeared to provide no protection against aerosols, regardless of the material they was made from (cotton or gauze) [18]. O’Kelly et al. documented that the average filtration efficiency of single layer fabrics was found to be 35% [19]. To obtain satisfactory filtration performance, fabric combinations or multilayer designs of woven masks are required [21,22].

KF masks are approved as sanitary aids (health masks) when their dust filtration efficiency reaches a certain standard level, and thus only approved masks can advertise their filtration performance against fine dusts [13]. To obtain the KF94 mask certification label, the ability of the mask to filter particulates must be tested using NaCl and paraffin oil for 30 s [13,23], conditions different from those used in our experiments. Even considering these operating differences, one of the KF94 masks (category C) exhibited low filtration capability against fine dusts as described above. It is necessary to periodically evaluate the filtering performances of the masks. Nevertheless, all the non-woven masks proved to be more effective than the woven masks at filtering PMs (Figure 2).

### 3.3. Filtration Efficiencies of the Masks against Airborne Droplets Containing Bacteria

**Figure 3:**
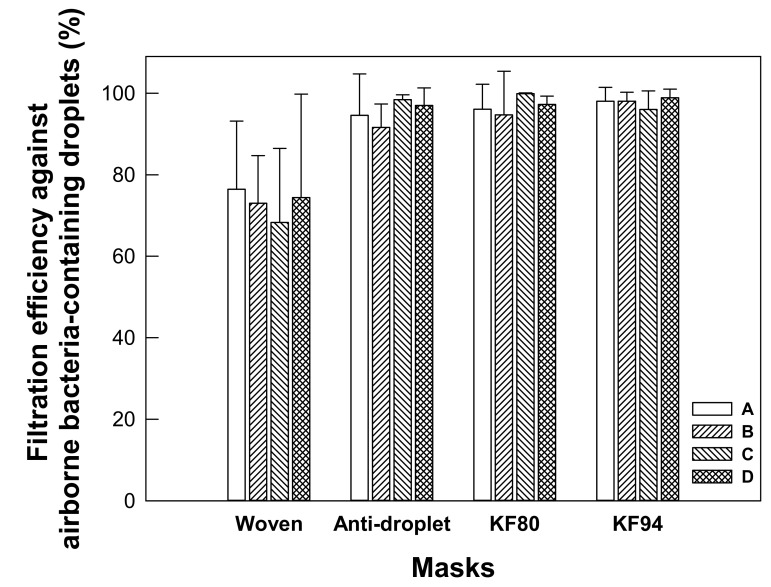
Filtration performances of commercially available face masks against airborne bacteria-containing droplets (*n* = 3). A, B, C, and D indicate categories of the masks described in Table S1.

The woven masks showed low average filtration efficiency levels of 68.3 ± 18.2 to 76.5 ± 16.7% against airborne bacteria-containing droplets (Figure 3). Non-woven masks, in contrast, showed high average filtration efficiency levels, specifically of 91.6 ± 5.7 to 97.0 ± 4.3 for the antidroplet masks, 96.1 ± 6.3 to 99.9 ± 0.2 for the KF80 masks, and 96.0 ± 4.5 to 98.9 ± 2.1 for the KF94 masks. Interestingly, category C of the KF94 mask achieved a high 96.0 ± 4.5% filtration efficiency level against the airborne bacteria-containing droplets, despite it having showed relatively low levels of 75.6 ± 8.3 to 77.6 ± 7.2% for filtering PMs as described above (Figure 2d and Figure 3). Overall, non-woven masks showed lower variations of filtration efficiency than did the woven masks.

**Figure 4:**
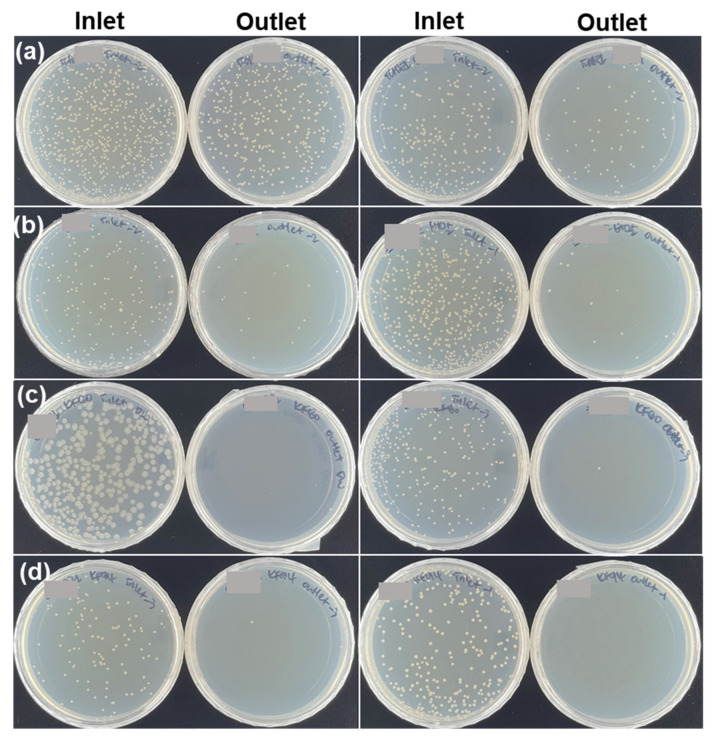
Photographs of collections of airborne bacteria-containing droplets on agar plates that had been placed near the inlet (first and third rows) and outlet (second and fourth rows) of the filtration unit containing (**a**) woven, (**b**) antidroplet, (**c**) KF80, and (**d**) KF94 masks, respectively.

Woven masks showed inferior filtering against airborne bacteria-containing droplets. The penetrated microdroplets were observed in the plates that had been placed near the outlet of our filtration unit (Figure 4a). This result showed the distinct possibility that users of such masks would inhale airborne bacteria. On the other hand, the tested non-woven masks were capable of effectively filtering the airborne bacteria-containing droplets (Figure 4b–d). It is worth noting that all of the masks, even the woven masks, were found to be effective at preventing a spray of airborne bacteria culture. As shown in Figure S3, no large droplets of the splashed airborne bacteria culture were collected from the plates located near the outlet of the filtration unit, indicating that such spray was incapable of penetrating the masks. The results suggested that wearing any of these masks would be effective at preventing the transmission of saliva derived from speaking, sneezing or coughing.

**Figure 5:**
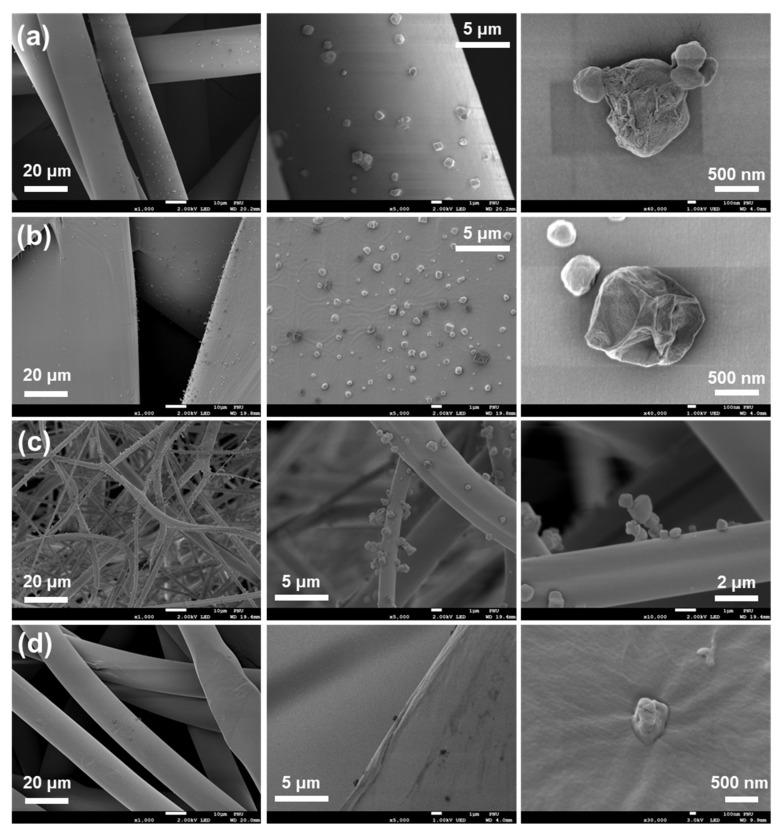
Scanning electron microscopy (SEM) images of a KF94 mask after it was used to filter airborne droplets containing bacteria. (**a**) Outer, (**b**) support, (**c**) filter, and (**d**) inner layers.

Filtered airborne bacteria-containing droplets were found in all of the layers of the KF94 mask, but were mostly filtered in the outer, support, and filter layers (Figure 5). Notably, a pile of particles was attached to the surface of filter fibers. EDS analysis indicated that the particles, having dimensions of 0.3–2.5 μm, were composed of C, Na, Cl, O, P, and N elements (Figure S4). The elemental mapping images thus demonstrated that the filtered particles contained the elements common in biological creatures such as bacteria and were thus attributable to the airborne bacteria-containing droplets.

**Figure 6:**
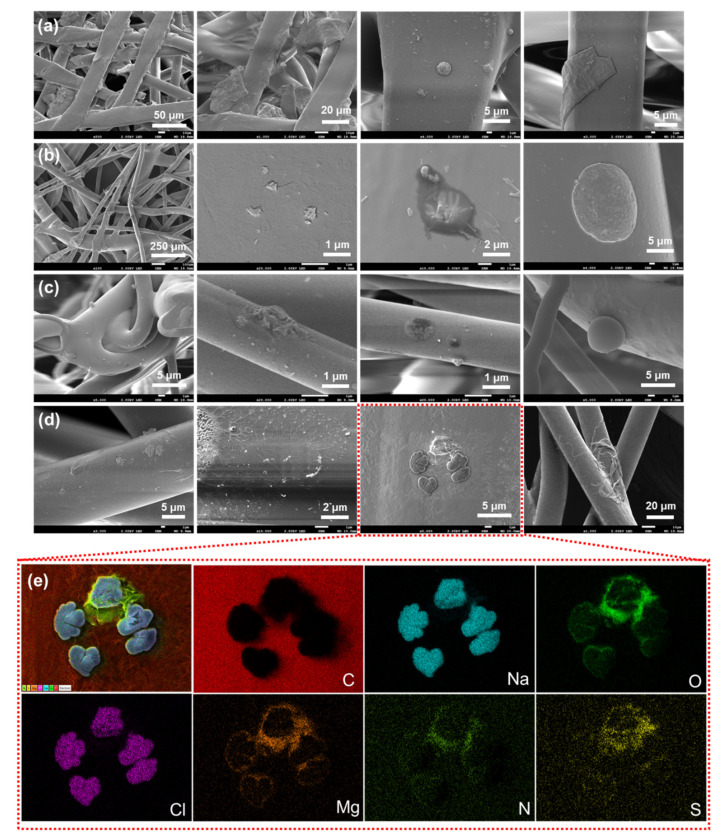
Scanning electron microscopy (SEM) images of a KF94 mask after it was subjected to actual use for a week. (**a**) Outer, (**b**) support, (**c**) filter, and (**d**) inner layers. (**e**) Energy-dispersive X-ray spectroscopy (EDS) elemental mapping images of the cloverlike particles collected in the mask after use.

The respective surfaces of four KF94 masks were investigated after these masks were used in actual practice for a week (Figure 6). Particles displaying spherical, rounded, square, fiberlike, and irregular shapes were identified, and were likely to be fine dust, saliva, and airborne bacteria-containing droplets. Clover-shaped particles were also observed, and EDS analysis of these particles showed that they were composed of C, Na, O, Cl, Mg, N, and S elements (Figure 6e), i.e., a composition similar to that of airborne bacteria-containing droplets (Figure S4). The results suggested that the tested masks would be suitable for preventing airborne droplet transmission in real-life use.

Our results were similar to those of other studies, in which KF94 provided efficient filtration while antidroplet masks achieved only ~80% filtration efficiency [9,24,25,26]. Jeong et al. found that the average filtration efficiencies of KF94 were higher than 95% against droplets containing *Staphylococcus epidermidis* (*S. epidermidis*) and those containing *Escherichia coli* (*E. coli*), while surgical masks showed filtration efficiency levels of more than 80% against these droplets [9]. Milton et al. reported that surgical masks effectively filtered viral aerosol droplets larger than 5 μm, but were less effective against small droplets [24]. Kim et al. documented that surgical masks were ineffective in filtering viral particles against coughing patients with SARS-CoV-2 infection [26]. Overall, non-woven masks achieved greater filtration efficiency levels against airborne droplets containing bacteria than did woven masks, and did so with stable performances.

### 3.4. Correlation of the Filtration Efficiency Levels of Masks against Particulate Matters (PMs) with the Efficiency Levels against Airborne Bacteria-Containing Droplets

**Figure 7:**
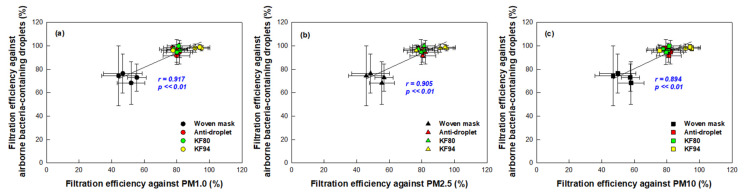
Pearson correlations of the filtration efficiencies of masks against particulate matters (PMs) versus their filtration efficiencies against airborne bacteria-containing droplets. (**a**) PM1.0 versus airborne bacteria-containing droplets. (**b**) PM2.5 versus airborne bacteria-containing droplets. (**c**) PM10 versus airborne bacteria-containing droplets.

The filtration performances of the masks against airborne droplets containing bacteria were found to be related to their filtration efficiencies against PMs, showing a significant positive linear correlation between them (Figure 7). Different pairs of variables showed different Pearson correlation coefficients. We measured Pearson coefficients of 0.905 (*p*-value < 0.01) between PM2.5 and airborne bacteria-containing droplets and 0.894 (*p*-value < 0.01) between PM10 and airborne bacteria-containing droplets (Figure 7b–c). The highest Pearson correlation coefficient, namely an *r* of 0.917 with a *p*-value < 0.01, was found between PM1.0 and airborne bacteria-containing droplets (Figure 7a). This high *r* was attributed to the dimensions of the particles of the airborne bacteria-containing droplets being similar to those of PM1.0, i.e., mostly less than 1 μm (Figure 5).

Antidroplet masks have flat shapes to fit loosely on the face (Table S1), and are not designed to block small airborne particles [27]. Unlike the cases in the US and Europe, there is no standard to determine the filtration efficiency of an antidroplet mask in Korea [1]. Fluid resistance to water is the requirement of an antidroplet mask [28]. Thus, it is difficult to choose the masks properly without confirmation of their performance for preventing the transmission of droplets. In this study, the filtration performances of the tested antidroplet masks were very similar to those of the tested KF80 masks (Figure 2b–c and Figure 3). Leung et al. reported that surgical face masks could efficiently reduce the transmission of viruses from infected individuals [29]. Our results also suggested that the antidroplet masks are acceptable alternatives if facepiece respirators are not available.

The approved PM-filtering masks were also able to effectively prevent transmission of airborne bacteria (Figure 2 and Figure 3). The KF mask has a cup-shaped design with a nose-wire that, relative to a planar design, can improve the fit of the mask on the face (Table S1). However, the ear-loop designs of KF masks are thought to be less effective in fitting than head-band designs [1,30]. Noti et al. reported that an improperly fitting N95 respiratory mask was much less effective at preventing the transmission of infectious viruses than was a well-fitting one [31]. Hill et al. also demonstrated that loosely fitting masks showed a greater-than-60% decrease in filtration efficiency against virion-sized particles compared to the ideal filtration efficiency [32]. Note that the filtration test in this study was carried out using the masks placed tightly into the filtration module (Figure 1a). Therefore, the filtration efficiency levels of the masks measured here should be considered the best that these masks can achieve, and fitting any such mask properly on one’s face is important to obtain the best possible filtration performance.

## 4. Conclusions

We evaluated the filtration performances of the commercially available masks with high market shares against PMs and airborne bacteria-containing droplets as a simulation of exposure to yellow dust and the transmission of airborne bacteria. The filtering capability of the masks against PMs showed a strong linear correlation with that against airborne droplets containing bacteria. This result indicated that a user can determine whether a mask is appropriate for preventing transmission of airborne bacteria based on its PM filtration efficiency.

We are all at risk of being exposed to infectious diseases through the transmission of airborne bacteria. Recently, the WHO strongly recommended using a mask to prevent the transmission of droplets. In this study, health masks (antidroplet, KF80, and KF94) achieved great filtering performances against airborne droplets containing bacteria as well as against PMs. The performances of the tested antidroplet masks appeared to be comparable to those of the tested KF80 masks. Our results indicated that wearing antidroplet, KF80, or KF94 masks will reduce airborne droplet transmission and provide adequate protection from bioaerosol particles generated by various possible activities such as breathing, speaking, coughing, and sneezing.

## Data Availability

Not applicable.

## Supplementary Materials

The following are available online at https://www.mdpi.com/article/10.3390/ijerph18157909/s1, Table S1: List of face masks tested in this study; Figure S1: Scanning electron microscopy (SEM) images of an unused woven mask; Figure S2: Scanning electron microscopy (SEM) images of a KF94 mask after it was used to filter air particulate matters (PMs); Figure S3: Photographs of collections of a spray of a *Bacillus subtilis* culture on agar plates from the inlet and outlet of filtration unit containing woven, antidroplet, KF80, and KF94 masks, respectively; Figure S4: Scanning electron microscopy (SEM) and energy-dispersive X-ray spectroscopy (EDS) elemental mapping images of a KF94 mask after it was used to filter airborne droplets containing bacteria.

## References

[1] Park S.H. (2020). Personal Protective Equipment for Healthcare Workers during the COVID-19 Pandemic. Infect. Chemother..

[2] World Helath Organization (WHO) WHO Coronavirus Disease (COVID-19) Dashboard (2021/02/15). https://covid19.who.int/.

[3] Muniyappa R., Gubbi S. (2020). COVID-19 pandemic, coronaviruses, and diabetes mellitus. Am. J. Physiol. Endocrinol. Metab..

[4] World Helath Organization (WHO) (2014). The WHO Guidelines Infection Prevention and Control of Epidemic and Pandemic-Prone Acute Respiratory Infections in Health Care.

[5] Siegel J.D., Rhinehart E., Jackson M., Chiarello L., the Healthcare Infection Control Practices Advisory Committee (2007). Guideline for Isolation Precautions: Preventing Transmission of Infectious Agents in Healthcare Settings.

[6] Otter J.A., Donskey C., Yezli S., Douthwaite S., Goldenberg S.D., Weber D.J. (2016). Transmission of SARS and MERS coronaviruses and influenza virus in healthcare settings: The possible role of dry surface contamination. J. Hosp. Infect..

[7] World Helath Organization Coronavirus Disease (COVID-19) Advice for the Public. https://www.who.int/emergencies/diseases/novel-coronavirus-2019/advice-for-public.

[8] United Stastes Environmental Protection Agency (USEPA) Particulate Matter (PM) Pollution. https://www.epa.gov/pm-pollution/particulate-matter-pm-basics.

[9] Jeong S.B., Ko H.S., Seo S.C., Jung J.H. (2019). Evaluation of filtration characteristics and microbial recovery rates of commercial filtering facepiece respirators against airborne bacterial particles. Sci. Total Environ..

[10] Eninger R.M., Honda T., Adhikari A., Heinonen-Tanski H., Reponen T., Grinshpun S.A. (2008). Filter performance of n99 and n95 facepiece respirators against viruses and ultrafine particles. Ann. Occup. Hyg..

[11] Lee S.A., Grinshpun S.A., Reponen T. (2008). Respiratory performance offered by N95 respirators and surgical masks: Human subject evaluation with NaCl aerosol representing bacterial and viral particle size range. Ann. Occup. Hyg..

[12] Brochot C., Saidi M.N., Bahloul A. (2020). How effective is the filtration of ’KN95’ filtering facepiece respirators during the COVID-19 pandemic?. Ann. Work Exp. Health.

[13] Korea Ministry of Food and Drug Safety (KMFDS) (2019). Guideline on Standards and Specifications for Filtering Respirators (for Industry).

[14] Ulrich N., Nagler K., Laue M., Cockell C.S., Setlow P., Moeller R. (2018). Experimental studies addressing the longevity of *Bacillus subtilis* spores The first data from a 500-year experiment. PLoS ONE.

[15] Chada V.G., Sanstad E.A., Wang R., Driks A. (2003). Morphogenesis of *Bacillus* spore surfaces. J. Bacteriol..

[16] Jung M.R., Horgen F.D., Orski S.V., Rodriguez C.V., Beers K.L., Balazs G.H., Jones T.T., Work T.M., Brignac K.C., Royer S.J. (2018). Validation of ATR FT-IR to identify polymers of plastic marine debris, including those ingested by marine organisms. Mar. Pollut. Bull..

[17] Rengasamy S., Eimer B., Shaffer R.E. (2010). Simple respiratory protection--evaluation of the filtration performance of cloth masks and common fabric materials against 20-1000 nm size particles. Ann. Occup. Hyg..

[18] Jung H., Kim J.K., Lee S., Lee J., Kim J., Tsai P., Yoon C. (2014). Comparison of filtration efficiency and pressure drop in anti-yellow sand masks, quarantine masks, medical masks, general masks, and handkerchiefs. Aerosol. Air Qual. Res..

[19] O’Kelly E., Pirog S., Ward J., Clarkson P.J. (2020). Ability of fabric face mask materials to filter ultrafine particles at coughing velocity. BMJ Open.

[20] Tcharkhtchi A., Abbasnezhad N., Zarbini Seydani M., Zirak N., Farzaneh S., Shirinbayan M. (2021). An overview of filtration efficiency through the masks: Mechanisms of the aerosols penetration. Bioact. Mater..

[21] Clase C.M., Fu E.L., Ashur A., Beale R.C.L., Clase I.A., Dolovich M.B., Jardine M.J., Joseph M., Kansiime G., Mann J.F.E. (2020). Forgotten technology in the COVID-19 pandemic: Filtration properties of cloth and cloth masks-A narrative Rreview. Mayo Clin. Proc..

[22] Lustig S.R., Biswakarma J.J.H., Rana D., Tilford S.H., Hu W., Su M., Rosenblatt M.S. (2020). Effectiveness of common fabrics to block aqueous aerosols of virus-like nanoparticles. ACS Nano.

[23] Han D.-H. (2015). Usage of Filtering-facepiece masks for healthcare workers and importance of fit testing. J. Korean Soc. Occup. Environ. Hyg..

[24] Milton D.K., Fabian M.P., Cowling B.J., Grantham M.L., McDevitt J.J. (2013). Influenza virus aerosols in human exhaled breath: Particle size, culturability, and effect of surgical masks. PLoS Pathog..

[25] Johnson D.F., Druce J.D., Birch C., Grayson M.L. (2009). A quantitative assessment of the efficacy of surgical and N95 masks to filter influenza virus in patients with acute influenza infection. Clin. Infect. Dis..

[26] Kim M.C., Bae S., Kim J.Y., Park S.Y., Lim J.S., Sung M., Kim S.H. (2020). Effectiveness of surgical, KF94, and N95 respirator masks in blocking SARS-CoV-2: A controlled comparison in 7 patients. Infect. Dis..

[27] Occupational Safety and Health Administration (OSHA) (2015). Hospital Respiratory Protection Program Toolkit: Resources for Respirator Program Administrators.

[28] National Institute of Food and Drug Safety Evaluation (NIFDS) (2016). Guideline on Establishment of Test Item in Preparation of Standards and Analytical Methods of Quasi-Drugs.

[29] Leung N.H.L., Chu D.K.W., Shiu E.Y.C., Chan K.H., McDevitt J.J., Hau B.J.P., Yen H.L., Li Y., Ip D.K.M., Peiris J.S.M. (2020). Respiratory virus shedding in exhaled breath and efficacy of face masks. Nat. Med..

[30] Centers for Disease Conrol and Prevention (CDC), The National Personal Protective Technology Laboratory (NPPTL) (2021). NPPTL Respirator Assessments to Support the COVID19 Response.

[31] Noti J.D., Lindsley W.G., Blachere F.M., Cao G., Kashon M.L., Thewlis R.E., McMillen C.M., King W.P., Szalajda J.V., Beezhold D.H. (2012). Detection of infectious influenza virus in cough aerosols generated in a simulated patient examination room. Clin. Infect. Dis..

[32] Hill W.C., Hull M.S., MacCuspie R.I. (2020). Testing of commercial masks and respirators and cotton mask insert materials using SARS-CoV-2 virion-sized particulates: Comparison of ideal aerosol filtration efficiency versus fitted filtration efficiency. Nano Lett..

