# Supplementary material for: Performance Evaluation of Commercially Available Masks in Korea for Filtering Airborne Droplets Containing Bacteria
Source: Int J Environ Res Public Health. 2021 Jul 26;18(15):7909. doi: 10.3390/ijerph18157909 (PMC8345791; doi:10.3390/ijerph18157909)
Supplement: Supplementary file 1 [file ijerph-18-07909-s001.zip › ijerph-1311793-supplementary.pdf]

[Supplementary Information]

Performance evaluation of commercially available masks in Korea for filtering airborne droplets containing bacteria

Eun-Hee Lee[1,*], Seung-Woo Lee[2], Seon Young Moon[3], Jangyup Son[4, 5]

[1] *Department of Microbiology, Pusan National University, 2 Busandaehak-ro 63 beon-gil, Geumjeong-gu, Busan, 46241, Republic of Korea*

[2] *Department of Fine Chemistry, Seoul National University of Science and Technology, Seoul, 01811, Republic of Korea*

[3] *AT-men Laboratory Co., Ltd, 305 Ho, Jinyang Bldg., 47, Kyonggidae-ro, Seodaemun-gu, Seoul, 03752, Republic of Korea*

[4] *Functional Composite Materials Research Center, Korea Institute of Science and Technology (KIST), Wanju-gun, Jeonbuk, 55324, Republic of Korea*

[5] *Division of Nano and Information Technology, KIST School University of Science and Technology (UST), Wanju-gun, Jeonbuk, 55324, Republic of Korea*

Corresponding author:

E-mail address: leeeh@pusan.ac.kr (E.-H. Lee)

Table S1. List of face masks tested in this study.

Materials	Type	Category	Shape	Number of Layers
Woven mask		A	Cup[a]	1
		B	Cup	1
		C	Cup	1
		D	Cup	1
Non-woven mask	Anti-droplet	A	Flat[b]	3 (Outer-filter-inner layers)[c]
		B	Flat	3 (Outer-filter-inner layers)
		C	Flat	3 (Outer-filter-inner layers)
		D	Flat	3 (Outer-filter-inner layers)
	KF80	A	Cup	4 (Outer-support-filter-inner layers)
		B	Cup	4 (Outer-support-filter-inner layers)
		C	Cup	3 (Outer-filter-inner layers)
		D	Cup	4 (Outer-filter-support-inner layers)
	KF94	A	Cup	3 (Outer-filter-inner layers)
		B	Cup	4 (Outer-filter-inner layers)
		C	Cup	4 (Outer-support-filter-inner layers)
		D	Cup	3 (Outer-filter-inner layers)

[a]Cup

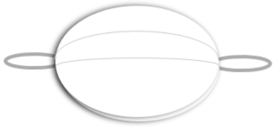

[b]Flat

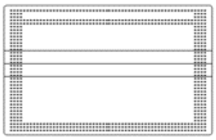

[c] Order of layers

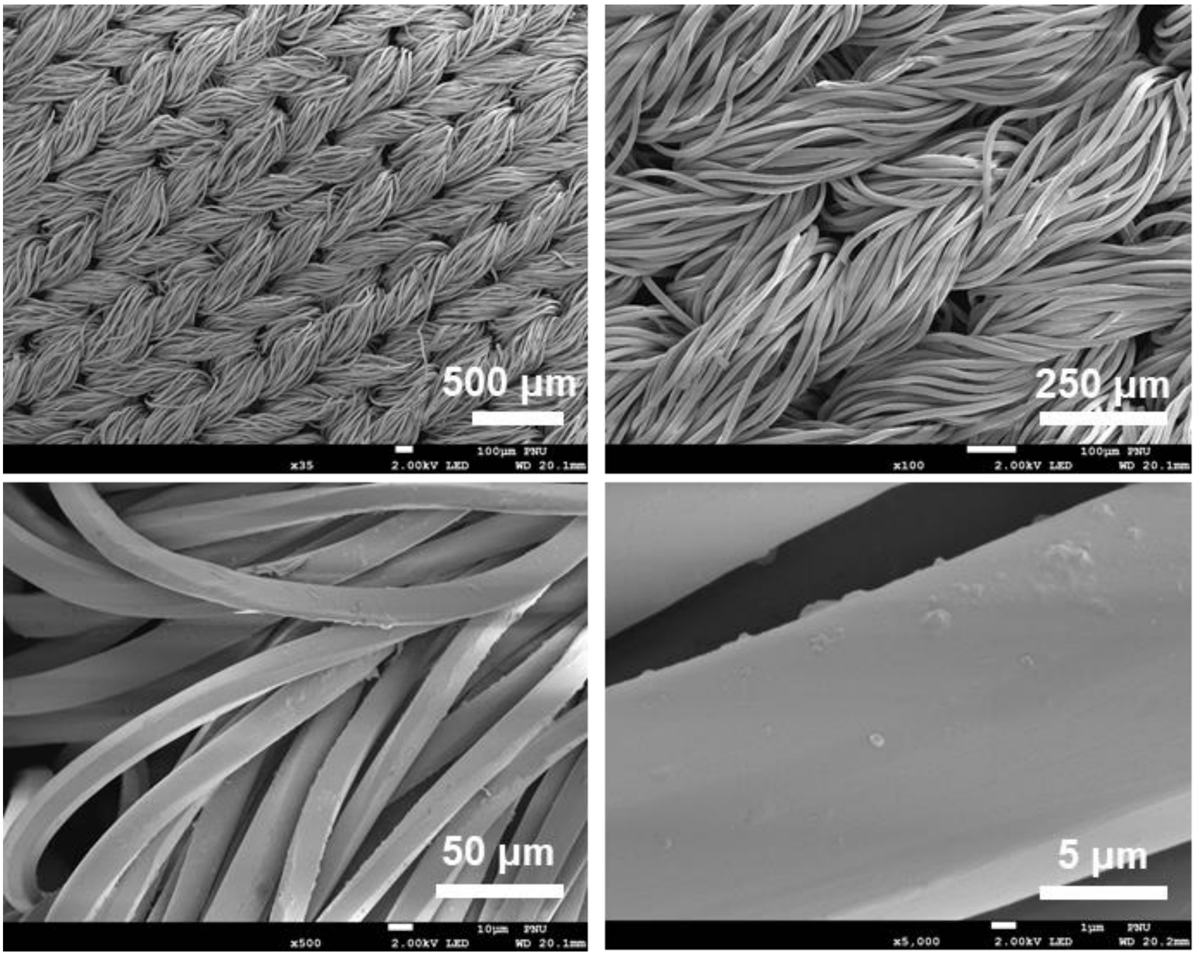

Figure S1. Scanning electron microscopy (SEM) images of an unused woven mask.

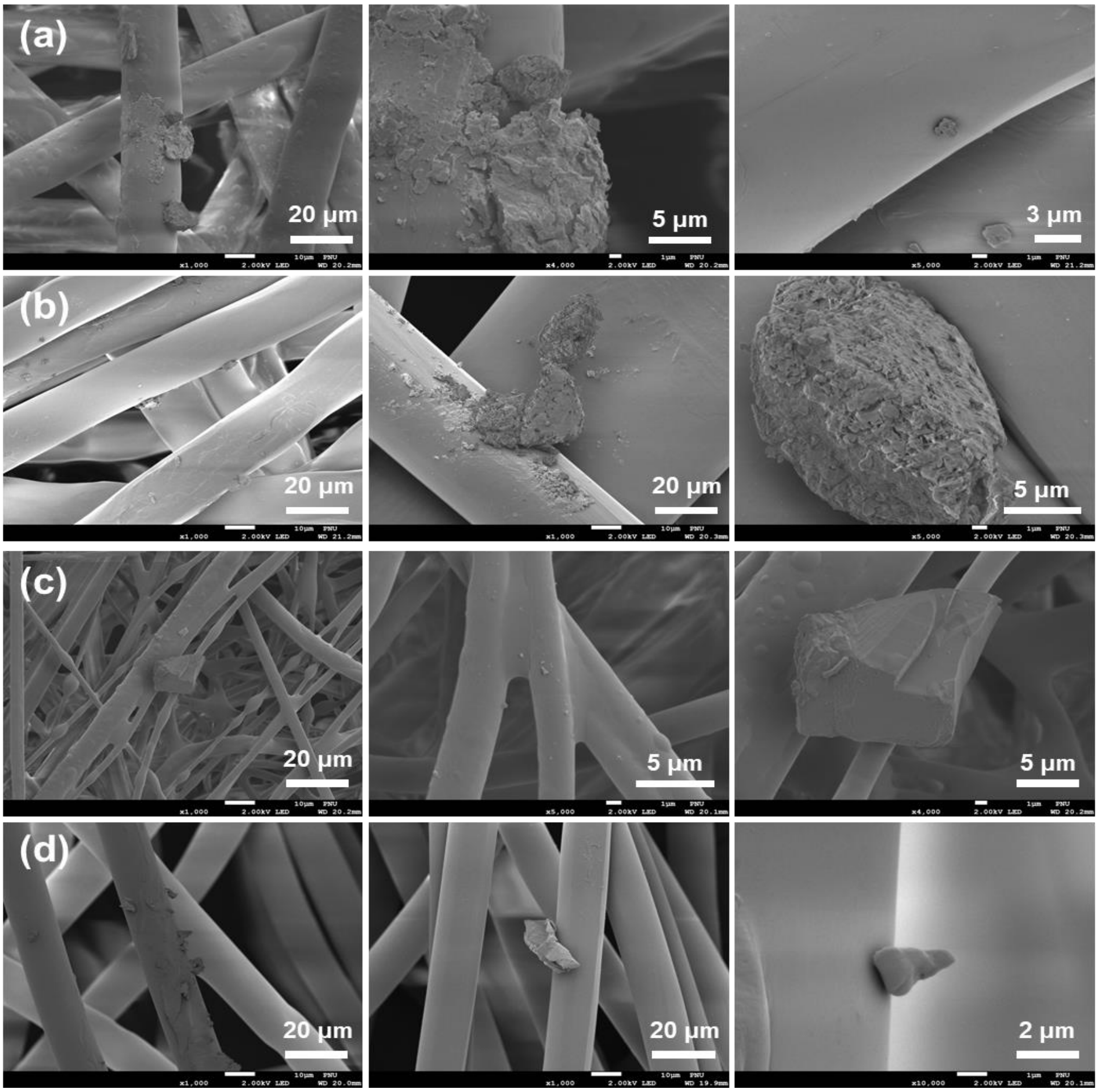

Figure S2. Scanning electron microscopy (SEM) images of a KF94 mask after it was used to filter air particulate matters (PMs). (a) Outer, (b) support, (c) filter, and (d) inner layers.

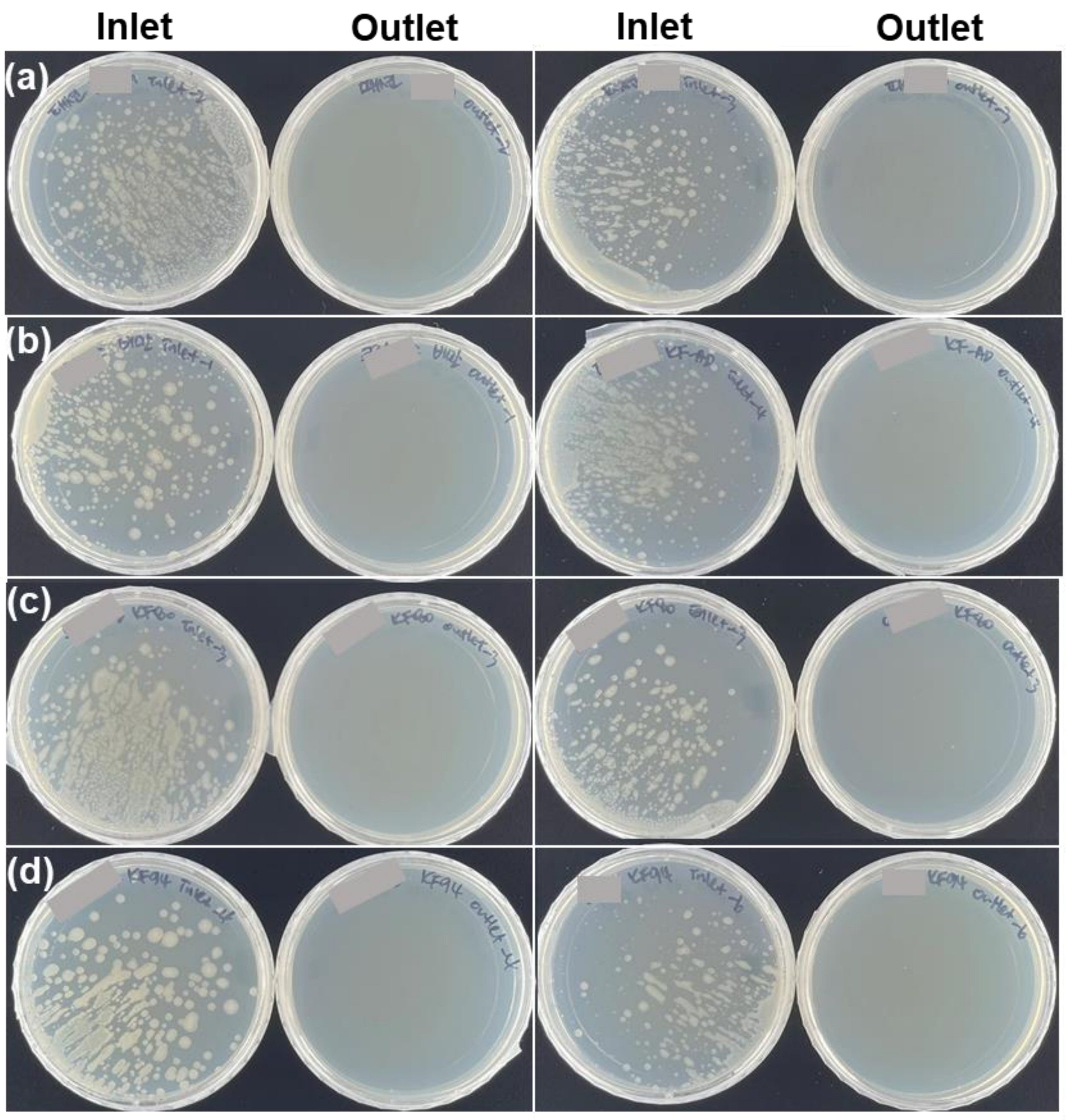

Figure S3. Photographs of collections of a spray of a *Bacillus subtilis* culture on agar plates from the inlet (first and third rows) and outlet (second and fourth rows) of filtration unit containing (a) woven, (b) anti-droplet, (c) KF80, and (d) KF94 masks, respectively.

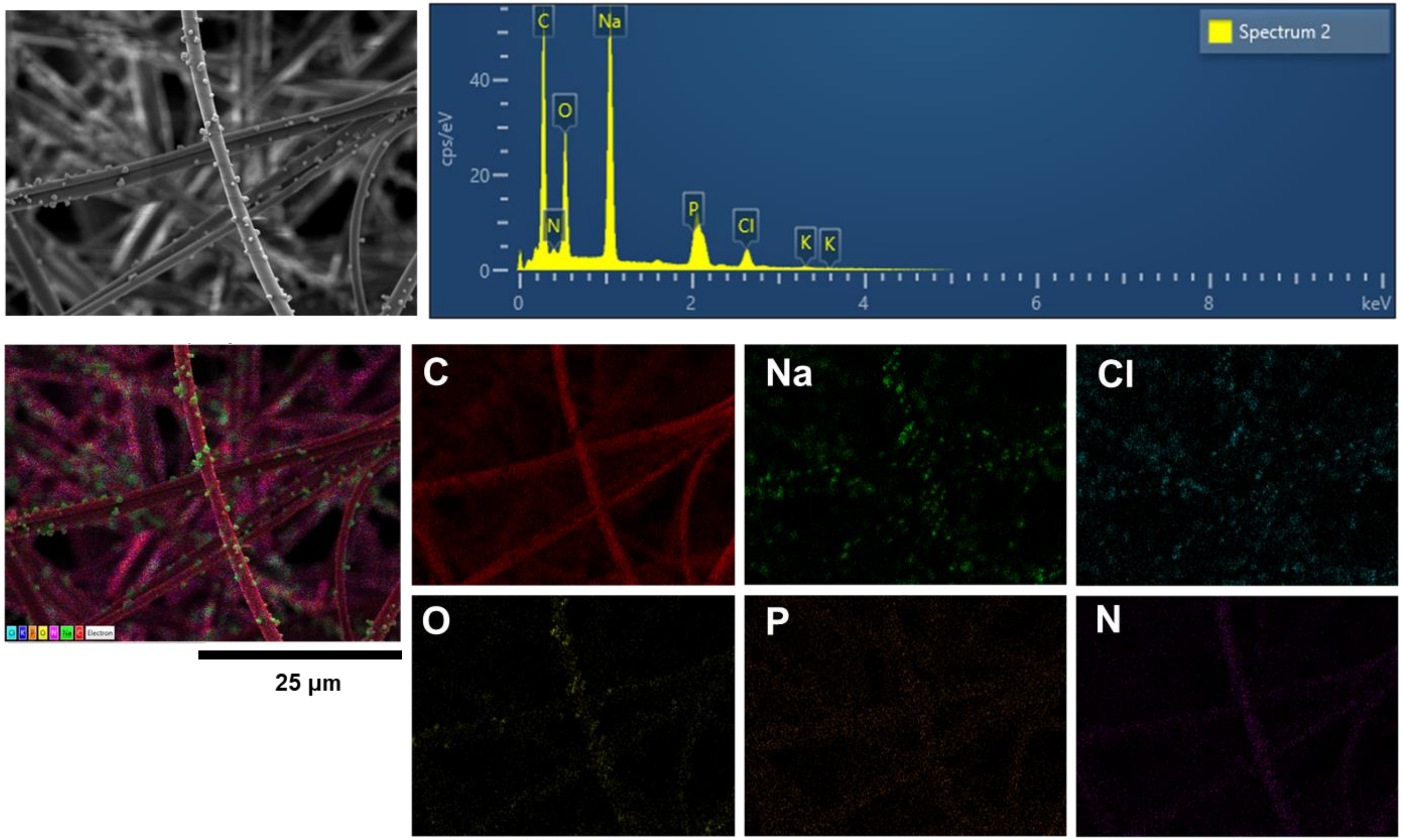

Figure S4. Scanning electron microscopy (SEM) and energy-dispersive X-ray spectroscopy (EDS) elemental mapping images of a KF94 mask after it was used to filter airborne droplets containing bacteria.
